# Enhancing Oxygen Evolution Catalysis by Tuning the Electronic Structure of NiFe-Layered Double Hydroxides Through Selenization

**DOI:** 10.3390/nano15040294

**Published:** 2025-02-14

**Authors:** Ze Wang, Yifang Liang, Taifu Fang, Xinyu Song, Luobai Yang, Liying Wen, Jinnong Wang, Dongye Zhao, Shifeng Wang

**Affiliations:** Key Laboratory of Plateau Oxygen and Living Environment of Xizang Autonomous Region, College of Science, Xizang University, Lhasa 850000, China; wangze@stu.utibet.edu.cn (Z.W.); lyf@utibet.edu.cn (Y.L.); fangtaifu@stu.utibet.edu.cn (T.F.); songxinyu@stu.utibet.edu.cn (X.S.); ylb18822178543@stu.utibet.edu.cn (L.Y.); wenliying@stu.utibet.edu.cn (L.W.); wangjinnong@stu.utibet.edu.cn (J.W.)

**Keywords:** electrocatalysis, water splitting, oxygen evolution reaction (OER), layered double hydroxides (LDHs), selenization

## Abstract

Electrocatalytic water splitting is a critical approach for achieving carbon neutrality, playing an essential role in clean energy conversion. However, the slow kinetics of the oxygen evolution reaction (OER) remains a major bottleneck hindering energy conversion efficiency. Although noble metal catalysts (e.g., IrO_2_ and RuO_2_) show excellent catalytic activity, their high cost and scarcity limit their applicability in large-scale industrial processes. In this study, we introduce a novel electrocatalyst based on selenized NiFe-layered double hydroxides (NiFe-LDHs), synthesized via a simple hydrothermal method. Its key innovation lies in the selenization process, during which Ni atoms lose electrons to form selenides, while selenium (Se) gains electrons. This leads to a significant increase in the concentration of high-valent metal ions, enhances electronic mobility, and improves the structural stability of the catalyst through the formation of Ni-Se bonds. Experimental results show that selenized NiFe-LDHs exhibit excellent electrocatalytic performance in 1 M KOH alkaline solution. In the oxygen evolution reaction (OER), the catalyst achieved an ultra-low overpotential of 286 mV at a current density of 10 mA cm⁻^2^, with a Tafel slope of 63.6 mV dec⁻^1^. After 60 h of continuous testing, the catalyst showed almost no degradation, far outperforming conventional catalysts. These results highlight the potential of NiFe-LDH@selenized catalysts in large-scale industrial water electrolysis applications, providing an effective solution for efficient and sustainable clean energy production.

## 1. Introduction

As the global demand for achieving carbon neutrality becomes increasingly urgent, developing efficient renewable energy technologies has become the top priority in the energy sector today [1,2]. Water splitting through electrocatalysis is widely recognized as a vital approach for clean energy conversion, consisting of two fundamental reactions: the oxygen evolution reaction (OER) and the hydrogen evolution reaction (HER) [3,4,5,6]. Owing to its complex multi-electron transfer mechanisms, the OER exhibits slow kinetics, which acts as a major hurdle in water splitting, substantially constraining the overall energy conversion efficiency [7,8]. While oxides of noble metals like IrO_2_ and RuO_2_ are known for their outstanding catalytic efficiency in OERs, their prohibitive cost and scarcity of resources significantly hinder their potential for large-scale use [9,10,11,12]. Therefore, finding cost-effective yet highly efficient alternative catalysts for OERs has become a critical challenge in the current research field.

Among the various candidate materials, transition metal oxides (TMOs) [13], metal–organic frameworks (MOFs) [14], transition metal sulfides (TMSs) [15], and transition metal phosphides (MPs) [16] have been widely applied in oxygen evolution reactions (OERs) due to their excellent catalytic performance, demonstrating different catalytic mechanisms and outstanding electrocatalytic efficiency. Despite the high catalytic activity of these materials in OERs, they still face a series of challenges, such as poor conductivity, low stability, and unclear catalytic mechanisms, which affect their long-term performance in practical applications [17]. In contrast, layered double hydroxides (LDHs), as non-precious metal electrocatalysts, have garnered widespread attention due to their unique layered structure and tunable chemical composition [18,19]. The tunable structure of LDHs offers ample opportunities for increasing active sites and optimizing catalytic performance, demonstrating high catalytic potential for the OER [20,21,22,23]. However, the inherent drawbacks of LDHs, particularly poor conductivity, inefficient charge transfer, and stability issues during prolonged electrochemical operation, still limit their further practical application [24,25,26,27]. Additionally, the limited surface area of active sites is insufficient to support an efficient OER, thus affecting the overall catalytic performance [28,29]. To address the limitations of LDHs in the oxygen evolution reaction (OER), researchers have employed various methods to enhance their conductivity and catalytic performance. For instance, Zhao et al. successfully synthesized a heterogeneous NiFe-LDH-P30 catalyst using an interface engineering strategy combined with O_2_ plasma treatment, significantly improving its catalytic performance [30]; additionally, Zhao et al. prepared Mo-doped NiFe-LDH through a hydrothermal method, further enhancing its intrinsic catalytic activity [31]. Although these studies have made significant progress in improving the conductivity and catalytic performance of LDHs, they still face issues such as insufficient material stability and poor conductivity, which limit their long-term application in OERs. Therefore, developing novel materials to overcome these drawbacks and enhance their stability and conductivity remains a key challenge in current research.

In this study, a NiFe-LDH@selenized electrocatalyst was successfully synthesized through a selenization modification strategy. The study found that after selenization, Ni atoms in NiFe-LDHs lose electrons, while Se atoms gain electrons, and this electron transfer process significantly promotes rapid electron transfer within the material. Meanwhile, the selenization reaction enhances the formation of metal–selenium bonds, further improving the structural stability of the material. Experimental results indicate that selenization treatment significantly enhanced the OER performance of NiFe-LDHs, with their catalytic activity clearly superior to that of pure NiFe-LDHs. This strategy not only effectively overcomes the inherent defects of LDHs, but also provides a new design approach for developing efficient, stable, and cost-effective OER catalysts, with significant theoretical importance and practical application potential.

## 2. Materials and Methods

### 2.1. Synthesis of NiFe-Layered Double Hydroxides and Their Derivatives

#### 2.1.1. Materials

Nickel nitrate hexahydrate (Ni(NO_3_)_2_·6H_2_O, analytical-grade) was purchased from Tianjin Damao Chemical Reagent Factory, while iron nitrate nonahydrate (Fe(NO_3_)_3_·9H_2_O, 99.99%), ammonium fluoride (NH_4_F, analytical-grade, 96%), urea (CO(NH_2_)_2_, analytical-grade, 99%), potassium hydroxide (KOH, 95%), and Nafion D-521 dispersion (5%) were provided by Shanghai Aladdin Biochemical Science and Technology Co., Ltd. (Shanghai, China). Selenium powder (Se, 99.9%) was purchased from Shanghai Macklin Biochemical Technology Co., Ltd. (Shanghai, China), and sodium borohydride (NaBH_4_, analytical-grade) was provided by Fuchen (Tianjin) Chemical Reagent Co., Ltd. (Tianjin, China). Absolute ethanol (C_2_H_5_OH, analytical-grade) was purchased from Chengdu Jinshan Chemical Reagent Co., Ltd. (Chengdu, China). All reagents used in the experiment were directly dissolved in deionized water, and no further purification of the chemical reagents and solvents was performed prior to use.

#### 2.1.2. Preparation Method of NiFe-LDH

The NiFe-LDH sample was prepared via a straightforward one-step hydrothermal process. Initially, 1.8 mmol of Ni(NO_3_)_2_·6H_2_O, 0.9 mmol of Fe(NO_3_)_3_·9H_2_O, 64 mmol of urea (CO(NH_2_)_2_), and 13.5 mmol of NH_4_F were accurately measured and dissolved in 50 mL of deionized water. The solution was stirred magnetically for 2 h to ensure complete dissolution and uniformity. The resulting mixture was then placed in a 100 mL stainless steel autoclave and subjected to a hydrothermal reaction at 105 °C for 12 h. After the reaction, the autoclave was allowed to cool to room temperature naturally. The product was then thoroughly washed with deionized water, collected via vacuum filtration, and dried in a vacuum oven at 60 °C for 12 h, resulting in the NiFe-LDH powder.

#### 2.1.3. Preparation Method of NiFe-LDH@selenized

NiFe-LDH@selenized was synthesized via a selenization process. Initially, varying amounts of selenium powder (50 mg, 100 mg, 150 mg, 200 mg, and 250 mg) were dissolved in 30 mL of deionized water containing 65 mg of sodium borohydride (NaBH_4_), and the solution was stirred to generate NaHSe. Subsequently, 0.2 g of pre-prepared NiFe-LDH was introduced into the NaHSe solution and stirred until a uniform mixture was achieved. The mixture was then transferred into a hydrothermal reactor and subjected to a reaction at 180 °C for 20 h. Once the reaction was complete, the reactor was cooled to room temperature naturally. The resulting product was thoroughly washed with deionized water and absolute ethanol, followed by drying at 60 °C for 12 h to obtain the NiFe-LDH@selenized composite.

### 2.2. Characterizations and Testing

#### 2.2.1. Characterizations

The crystalline phases of the samples were thoroughly analyzed using a Bruker D8 Advance X-ray diffractometer (Billerica, MA, USA), equipped with a Co Kα radiation source (λ = 1.79026 Å). Surface morphology was observed using a CIQTEK SEM5000 (Hefei, Anhui, China), while elemental distribution was examined with a Thermo Fisher Apreo C field emission scanning electron microscope (FE-SEM), combined with energy-dispersive X-ray spectroscopy (EDS), allowing for detailed visualization of both surface features and elemental mapping. The nanostructure was further investigated using an FEI Tecnai G2 F30 (Waltham, MA, USA) transmission electron microscope (TEM), providing high-resolution images. Additionally, the surface chemical composition and electronic structure of NiFe-LDH and its composites were studied using a Thermo Fisher Escalab 250XI (Waltham, MA, USA) X-ray photoelectron spectrometer (XPS), with a monochromatic Al Kα radiation source (hv = 1486.6 eV). The XPS measurements were conducted under a 150W power setting, with a beam size of 650 μm, a voltage of 14.8 kV, and a current of 1.6 A. Charge calibration was performed using the C 1s peak (284.8 eV) attributed to adventitious carbon.

#### 2.2.2. Electrocatalytic Performance Testing

In this study, the electrochemical properties during the OER were evaluated in a 1 M KOH solution using a three-electrode setup connected to an electrochemical workstation (CHI660E). The system comprised a glassy carbon as the working electrode, Hg/HgO as the reference electrode, and a graphite rod as the counter electrode. To ensure consistent and reliable data, all potentials were referenced to the reversible hydrogen electrode (RHE) by applying the conversion formula: E_RHE_ = E_Hg/HgO_ + 0.923 V. The current density (J) was calculated by dividing the current (I) by the geometric area (S) of the working electrode. The working electrode was a glassy carbon electrode with a diameter of 3 mm, so its geometric area (S) could be obtained using the formula for the area of a circular electrode, S= πr^2^, where r is the radius of the electrode. Initially, cyclic voltammetry (CV) was employed for system activation, using 20 cycles at a sweep rate of 0.1 V/s to stabilize the system and obtain reproducible voltammograms. Linear sweep voltammetry (LSV) was then carried out in the potential range of 0 to 0.8 V, at a scan rate of 5 mV/s, to assess the electrocatalytic activity. Additionally, electrochemical impedance spectroscopy (EIS) was employed to examine the electrochemical properties of the catalyst, with the frequency range varying from 100 kHz to 1 Hz, with an amplitude of 5 mV, and a test voltage selected at a current density of 10 mA/cm^2^.

## 3. Results

**Figure 1:**
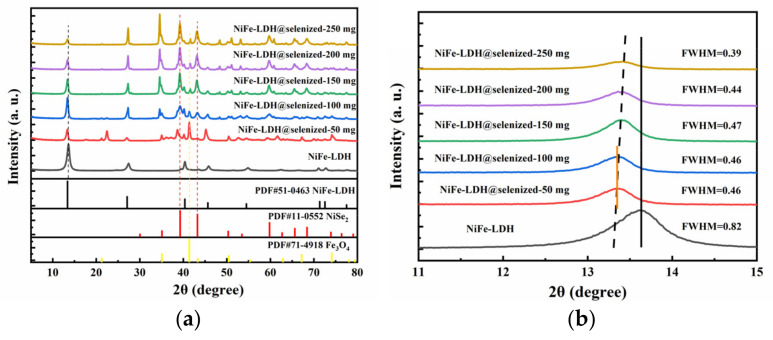
(**a**) XRD patterns of NiFe-LDH and NiFe-LDH@selenized; (**b**) magnified view of selected region.

The X-ray diffraction (XRD) pattern (Figure 1a) shows the crystal structure of NiFe-LDH and its selenized form, NiFe-LDH@selenized. The XRD pattern of NiFe-LDH shows five prominent diffraction peaks, located at 13.4°, 27.1°, 40.4°, 45.6°, and 54.5°, corresponding to the (003), (006), (012), (015), and (018) planes of NiFe-LDH (PDF#00-051-0463), confirming the successful preparation of NiFe-LDH. In the XRD pattern of the NiFe-LDH@selenized composite, as the amount of selenium powder increases, the characteristic peaks of NiFe-LDH remain unchanged, but the intensity of some diffraction peaks decreases, which may be due to the formation of new substances on the surface of NiFe-LDH after selenization. Further observation reveals that during the selenization process, two new substances, NiSe_2_ and Fe_3_O_4_, were generated on the surface of NiFe-LDH. The newly appeared diffraction peaks are located at 35.1°, 39.3°, 43.2°, 50.3°, and 59.8°, corresponding to the (200), (210), (211), (220), and (311) planes of NiSe_2_ (PDF#00-011-0552); in addition, there are new peaks at 2θ = 21.3°, 41.4°, and 67.2°, corresponding to the (111), (311), and (511) planes of Fe_3_O_4_ (PDF#01-071-4918). These results suggest that the formation of NiSe_2_ and Fe_3_O_4_ occurs on the surface of NiFe-LDH during selenization. As shown in Figure 1b, we magnified the (003) plane located at 13.4° and found that as the amount of selenium powder increased, the peak shifted in the negative direction, with the largest shift observed for the materials with 50 mg and 100 mg of selenium [32]. At the same time, the full width at half maximum (FWHM) decreased, indicating that the crystallinity increased with the amount of selenium powder. The calculated lattice constant a shows an increasing–decreasing trend, which may be related to the amount of selenium powder added and the interaction of the surface substances of NiFe-LDH.

**Figure 2:**
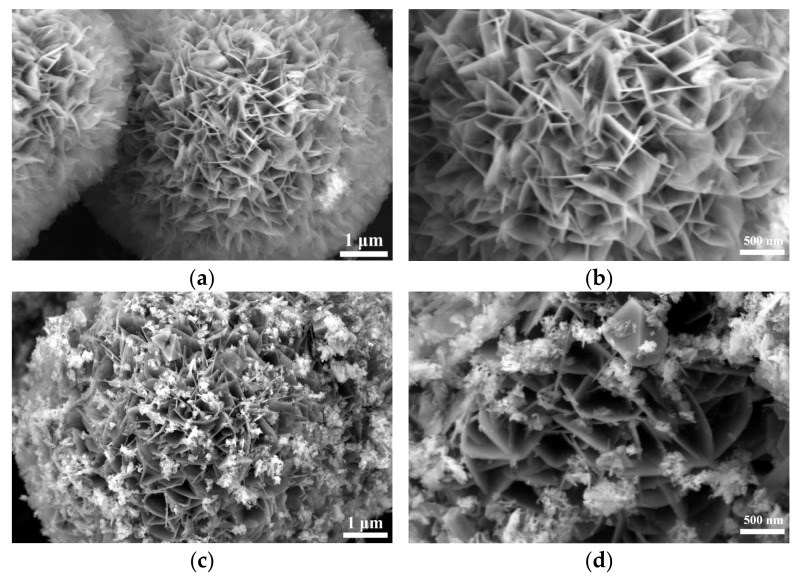

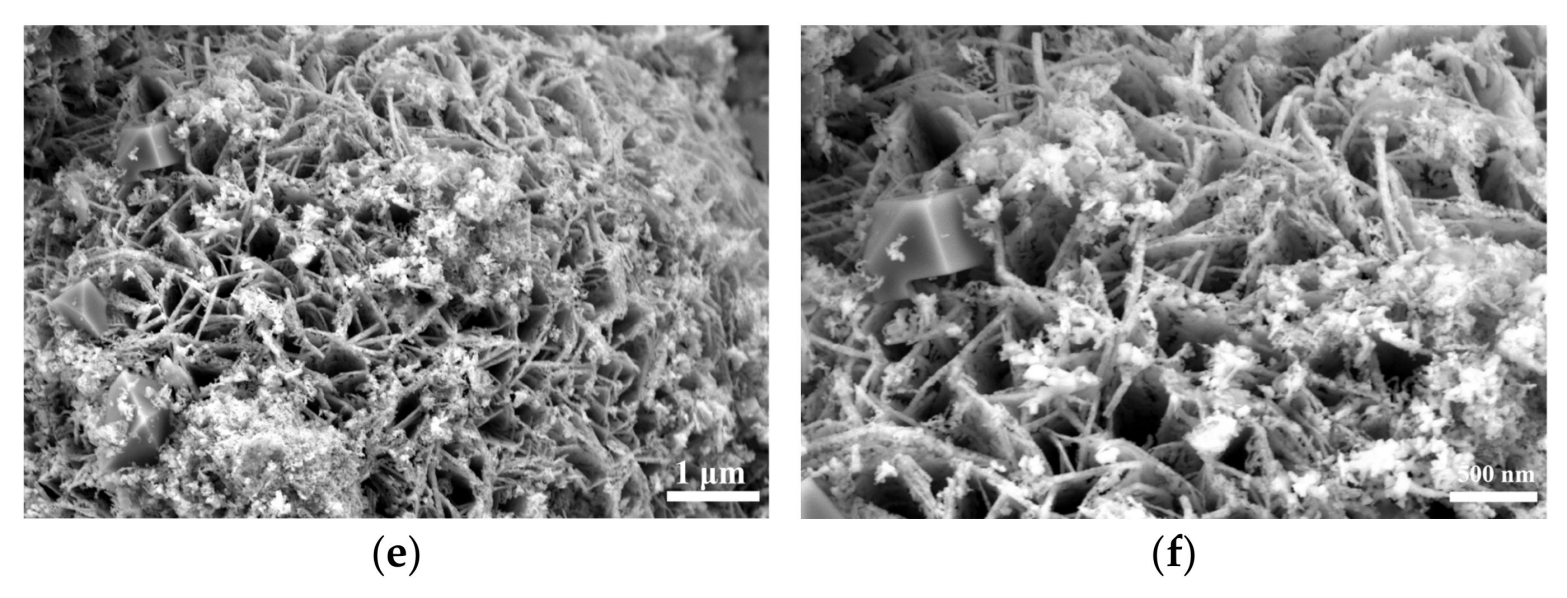
Scanning electron micrographs (SEMs) of NiFe-LDH and NiFe-LDH@selenized: (**a**,**b**) NiFe-LDH; (**c**,**d**) NiFe-LDH@selenized—100 mg; (**e**,**f**) NiFe-LDH@selenized—250 mg.

SEM analysis of Figure S1 and Figure 2 shows that the selenization process significantly altered the morphology of NiFe-LDH. The pristine NiFe-LDH exhibits a typical flower-like structure composed of nanosheets, which provides a large specific surface area for electrocatalytic reactions, enhancing catalytic activity. However, as the amount of selenium powder increases, the surface of the nanosheets gradually becomes covered by granular and blocky substances, causing the edges to blur and the flower-like structure to gradually disappear. Preliminary XRD analysis indicates that these coatings are primarily NiSe_2_ and Fe_3_O_4_, with the corresponding diffraction peak intensities increasing as the amount of selenium powder increases, while the characteristic diffraction peaks of NiFe-LDH significantly weaken. This phenomenon suggests that as the selenization reaction progresses, more NiSe_2_ and Fe_3_O_4_ phases gradually form on the surface of NiFe-LDH, and these newly formed substances occupy a larger surface area.

**Figure 3:**
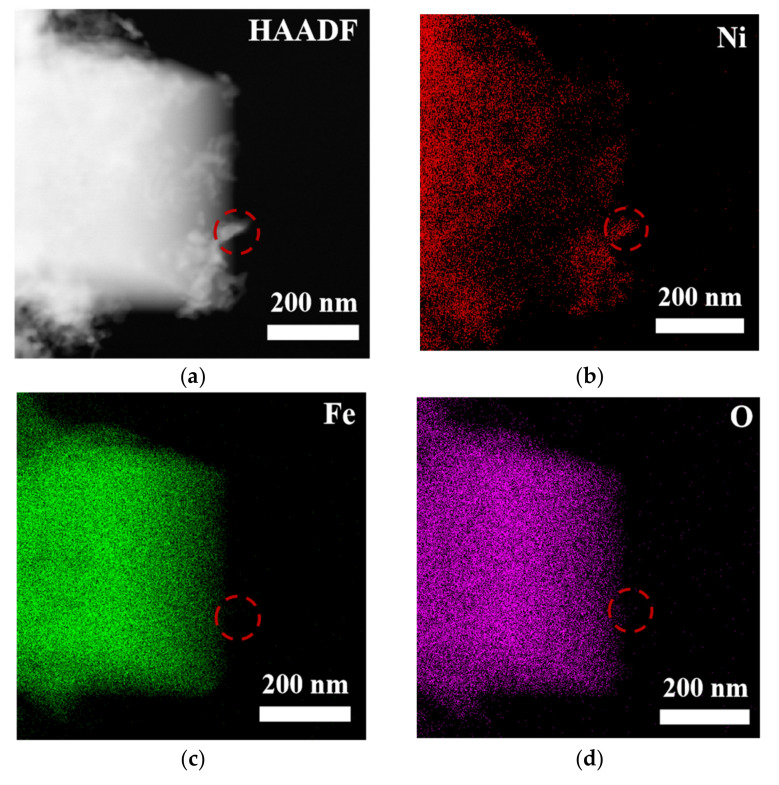

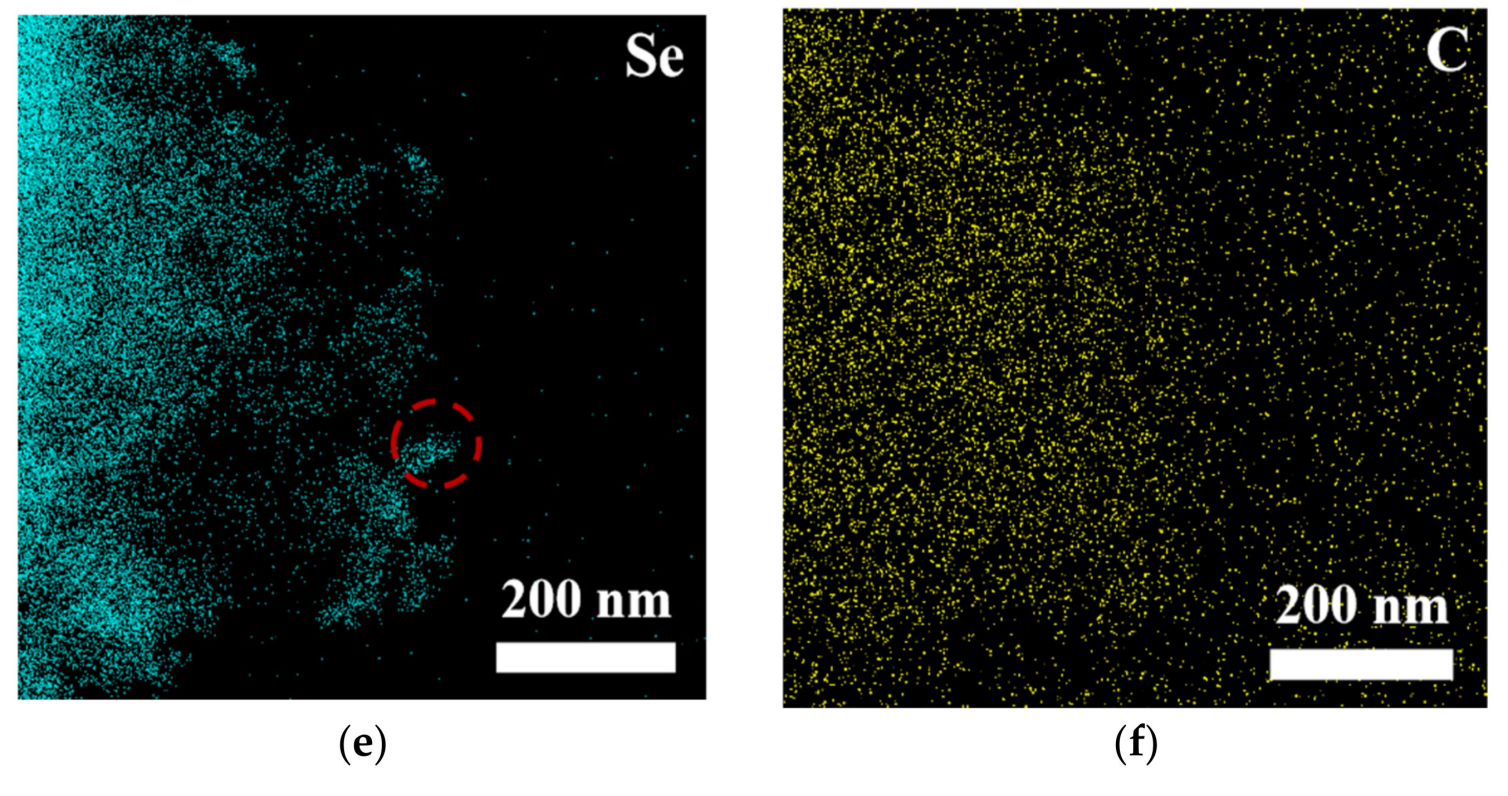
(**a**) HAADF image and (**b**–**f**) MAPPING image of NiFe-LDH@selenized-100 mg.

To comprehensively and systematically analyze the composition and microstructure of the material, this study employed scanning electron microscopy (SEM) and transmission electron microscopy (TEM), along with energy-dispersive X-ray spectroscopy (EDS), for detailed characterization of the samples (see Figures S2, S3 and Figure 3). The SEM-EDS analysis results (Figure S1) indicate that the elements nickel (Ni), iron (Fe), oxygen (O), selenium (Se), and carbon (C) were uniformly distributed in the sample, suggesting that the material exhibited good macroscopic uniformity, confirming the successful preparation of the multi-element composite material. Further TEM-EDS imaging analysis of the bulk and granular materials generated on the surface of NiFe-LDH (Figure 3) revealed that the edges of the bulk material were enriched with nickel and selenium, suggesting that these particles could be NiSe_2_, while the bulk region could be Fe_3_O_4_. TEM point-scanning analysis of this region (Figure S3) further validated this hypothesis, confirming that the granular material was NiSe_2_ and the bulk material was Fe_3_O_4_.

**Figure 4:**
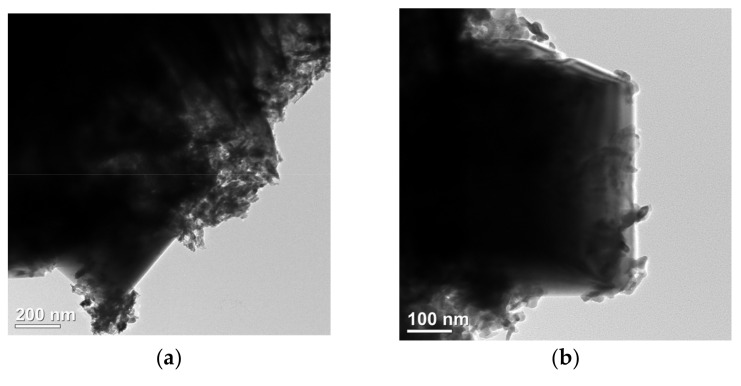

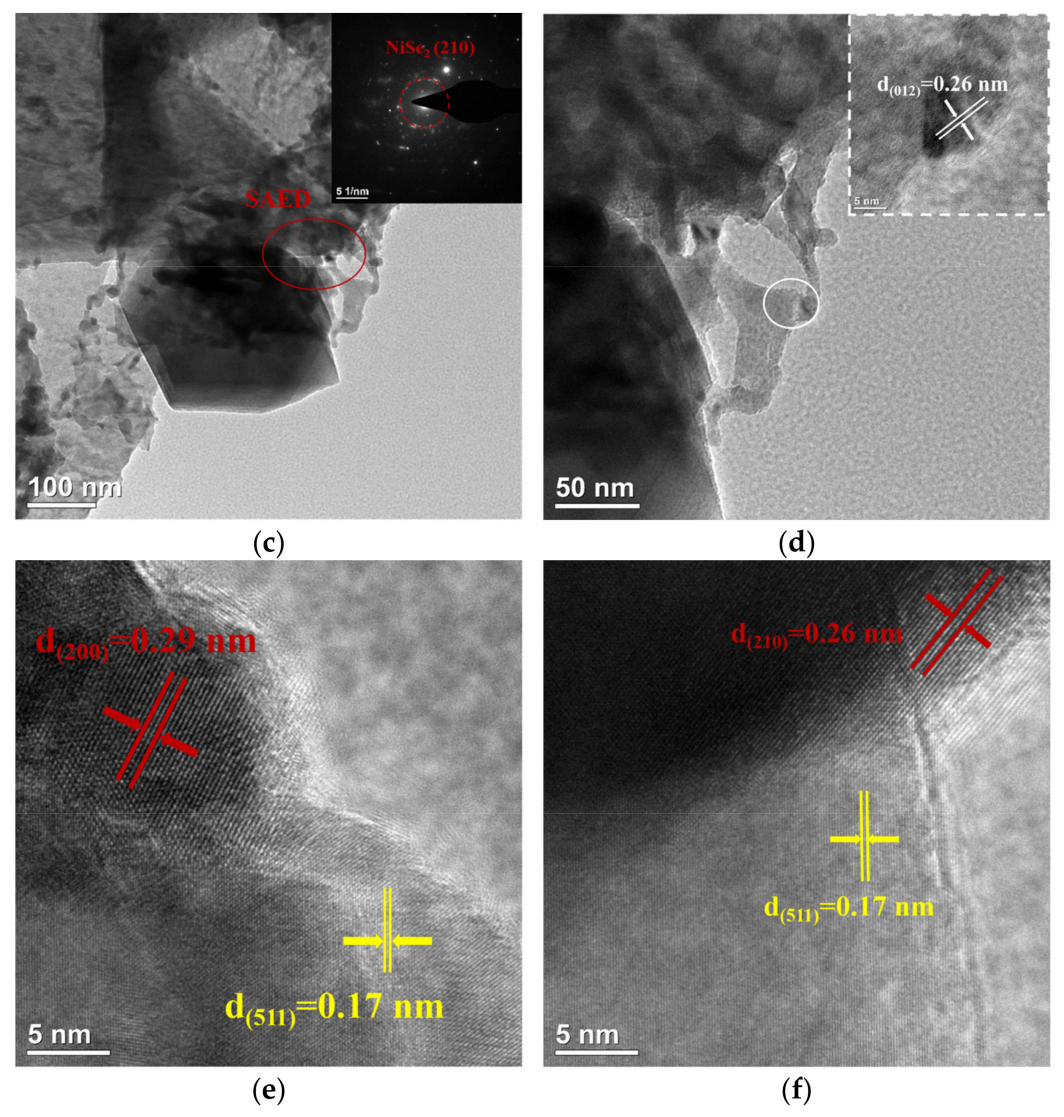
(**a**–**f**) HRTEM image of NiFe-LDH@selenized—100 mg.

The microstructure of NiFe-LDH@selenized-100 mg was systematically characterized using transmission electron microscopy (TEM) to investigate its morphology and interfacial properties (see Figure 4). Figure 4a presents a typical structure of the composite material, where layered, particulate, and bulk materials are intricately intertwined, forming a highly complex and ordered microstructure. This morphology reveals a tight integration between the multiphase materials, indicating strong interactions among different components. Figure 4b further illustrates a distinct heterogeneous interface between the particulate NiSe_2_ and Fe_3_O_4_ through high-resolution TEM images, clearly showcasing the coexistence of these two phases and their potential heterojunction effects. The selected area electron diffraction (SAED) pattern shown in Figure 4c confirms the (210) crystal plane in NiSe_2_, which is highly consistent with the X-ray diffraction (XRD) analysis results, validating the crystal structure of the NiSe_2_ phase. Additionally, the lattice fringe spacing of 0.26 nm shown in Figure 4d corresponds to the (012) crystal plane of NiFe-LDH. Further high-resolution TEM analysis (Figure 4e,f) reveals lattice fringe spacings of 0.29 nm, 0.26 nm, and 0.17 nm, corresponding to the (200) and (210) planes of NiSe_2_ and the (511) plane of Fe_3_O_4_, further confirming the successful synthesis of NiSe_2_ and Fe_3_O_4_. The clarity of these lattice fringes not only reveals high crystal quality, but also demonstrates the heterojunction characteristics between the two-phase materials. Although the TEM images do not directly reveal a clear heterogeneous interface between NiFe-LDH and NiSe_2_ and Fe_3_O_4_, the morphological analysis indicates their inter-relationship and loading behavior within the composite material. In summary, the selenization process effectively altered the internal electronic structure of NiFe-LDH, allowing it to successfully combine with Se to produce NiSe_2_ and Fe_3_O_4_. This transformation significantly enhanced the complexity and interactions of the material microstructure.

**Figure 5:**
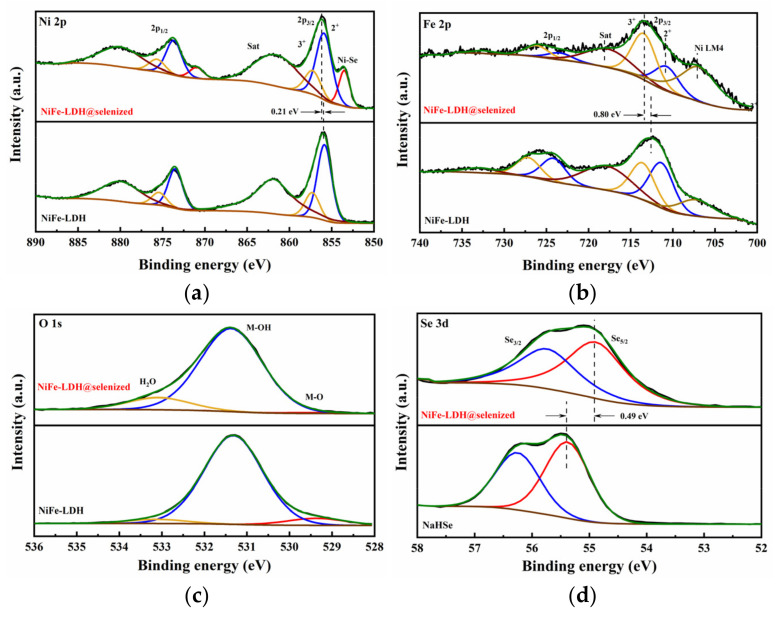
X-ray photoelectron spectra of (**a**) Ni 2p spectrum, (**b**) Fe 2p spectrum, and (**c**) O 1s spectrum for NiFe-LDH (**lower**) and NiFe-LDH@selenized-100 mg (**upper**); (**d**) Se 3d spectrum for NaHSe (**lower**) and NiFe-LDH@selenized-100 mg (**upper**).

Figure 5 presents the X-ray photoelectron spectroscopy (XPS) analysis results of NiFe-LDH, NiFe-LDH@selenized-100 mg, and NaHSe. The full XPS spectrum of NiFe-LDH@selenized-100 mg (see Figure S4) indicates that the composite material is primarily composed of Ni, Fe, O, C, and Se, which is consistent with previous energy-dispersive X-ray spectroscopy (EDS) results. Compared with NiFe-LDH, the Ni 2p region of NiFe-LDH@selenized-100 mg shows a new characteristic peak at 853.5 eV (Figure 5a), which is typically attributed to Ni-Se complexes. Combining literature data and XPS binding energy reference values, this peak is more likely to correspond to the characteristic signal of NiSe_2_, thereby indirectly confirming the formation of NiSe_2_ [33]. In Figure 5a, the Ni 2p spectrum of NiFe-LDH@selenized-100 mg shows peaks at 857.3 eV and 875.7 eV, corresponding to Ni^3^⁺, while peaks at 855.9 eV and 873.7 eV correspond to Ni^2^⁺. Compared to NiFe-LDH, the binding energy of the Ni 2p_3_/_2_ peak is shifted 0.21 eV toward higher energy, indicating that the selenization process increased the proportion of higher-valence Ni [34]. Figure 5b displays the Fe 2p spectrum; despite the influence of the Ni Auger peak on the fine spectrum of Fe, peak fitting analysis reveals that the peaks at 713.4 eV and 726.1 eV correspond to Fe^3^⁺, while those at 710.8 eV and 723.4 eV correspond to Fe^2^⁺. Compared to the unselenized sample, the binding energy of the Fe 2p_3/2_ orbital is also shifted toward higher energy, indicating an increased proportion of high-valent Fe in the material, further confirming that selenization affects the valence states of Fe^3^⁺ and Fe^2^⁺ [35,36]. This shift in binding energy is often closely related to the change in the position of the d-band center. A shift in the binding energy to higher energies indicates a downward movement of the d-band center, which may reduce the adsorption strength of oxygen intermediates (such as *OH, O, and OOH) on the material surface, thereby optimizing the reaction energy barrier in the OER process [37]. This adjustment in the electronic structure may be one of the key factors enabling NiFe-LDH@selenized-100 mg to exhibit higher OER activity. In the O 1s spectrum (Figure 5c), the characteristic peaks of NiFe-LDH@selenized-100 mg can be divided into three components, corresponding to metal–oxygen bonds (M-O, 529.4 eV), oxygen in OH⁻ groups (M-OH, 531.4 eV), and surface-adsorbed water (M-H_2_O, 533.1 eV) [35]. Figure 5d compares the Se 3d spectrum of NiFe-LDH@selenized-100 mg with the fine spectrum of Se in NaHSe, where the binding energy of Se 3d_5/2_ in NaHSe is located at 55.4 eV, likely corresponding to elemental or composite Se, consistent with the experimental results. However, in the composite material, the binding energy of Se 3d_5/2_ decreases from 55.4 eV to 54.9 eV, indicating that Se was reduced during the reaction by gaining electrons, corresponding to the formation of Ni-Se compounds. In summary, significant charge transfer occurs during the selenization process: Ni loses electrons to form selenides, while Se is reduced by gaining electrons. This charge transfer process not only confirms the role of selenization in modulating the electronic structure of NiFe-LDH@selenized—100 mg, but also provides theoretical support for the oxygen evolution reaction (OER) [38,39]. Specifically, during the OER process, the high-valent metal centers of Ni and Fe (such as Ni^3^⁺ and Fe^3^⁺) serve as active sites, facilitating the generation of oxygen molecules through interactions with water molecules or hydroxide ions in the solution. This process typically involves multiple steps, during which high-valent metal ions undergo reduction by absorbing electrons and drive the desorption of oxygen molecules [40]. Therefore, the selenization process effectively promotes the kinetics of the oxygen evolution reaction by optimizing the electronic structure of the catalyst, enhancing the overall catalytic efficiency of the composite material.

**Figure 6:**
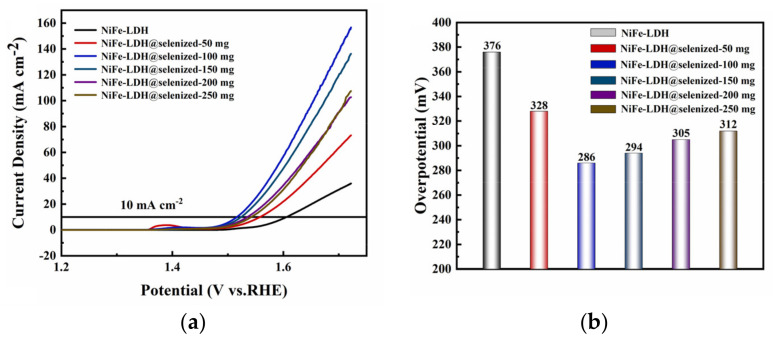

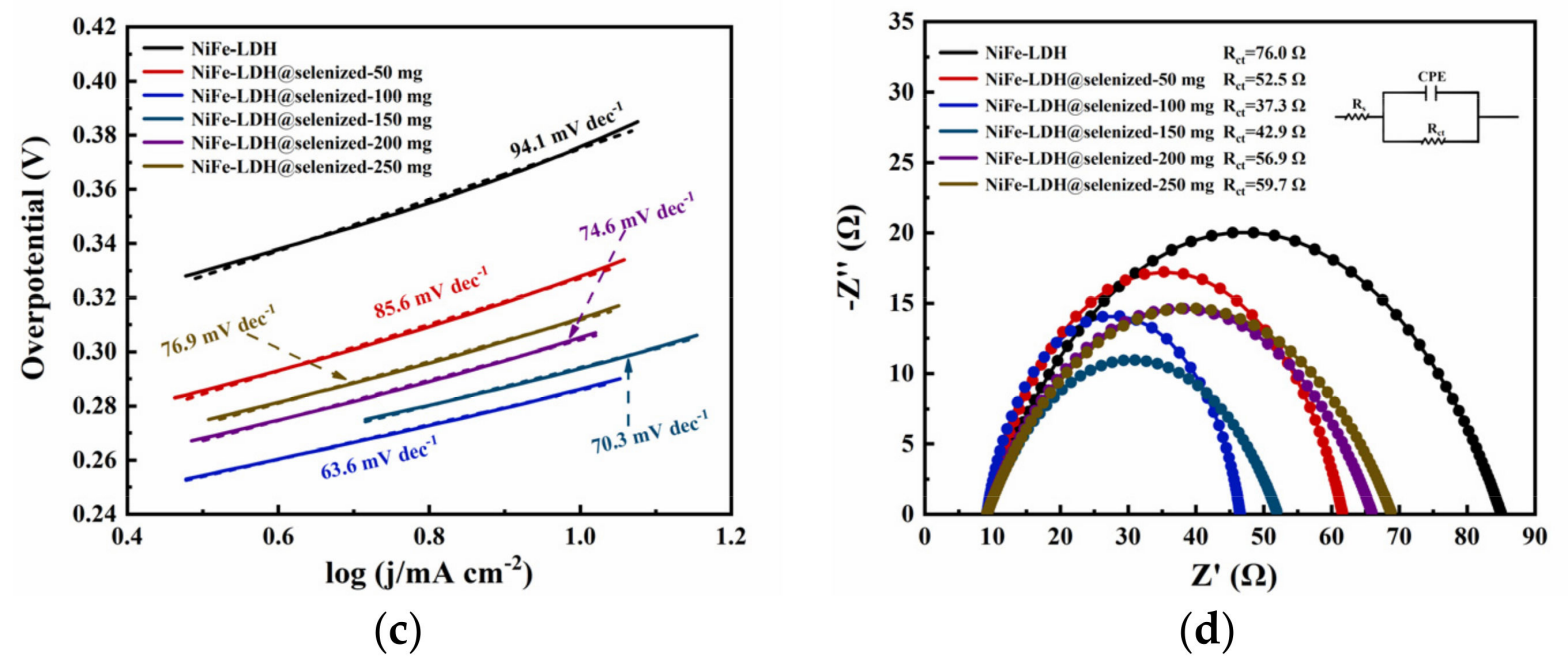
LSV curves (**a**), overpotential (**b**), Tafel slope (**c**), and electrochemical impedance plots (**d**) were measured in 1 M KOH alkaline solution.

**Figure 7:**
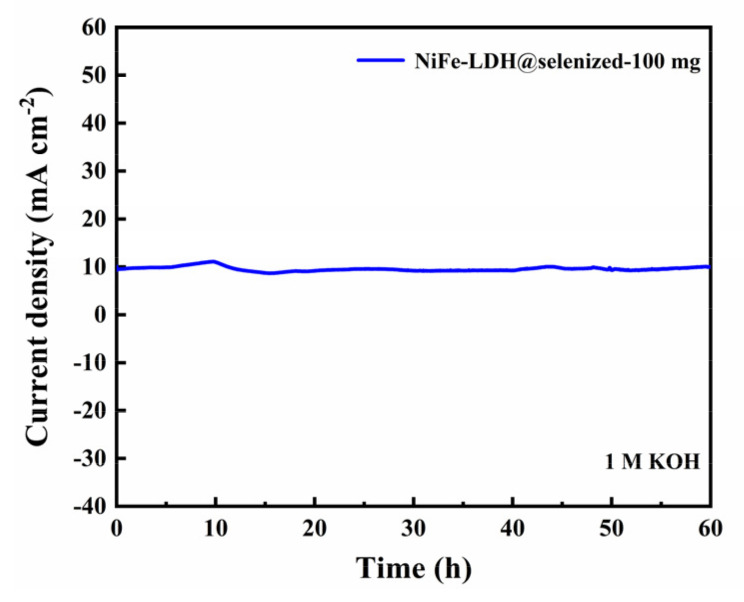
Chronopotentiometric curve of NiFe-LDH@selenized-100 mg at current density of 10 mA cm⁻^2^.

The electrocatalytic activity of the catalyst was evaluated by linear sweep voltammetry (LSV) to generate polarization curves, and the measurements were conducted in a 1 M KOH alkaline solution. At a current density of 10 mA/cm^2^, the potentials recorded under six different conditions were 1.606 V, 1.558 V, 1.516 V, 1.524 V, 1.535 V, and 1.542 V (Figure 6a), with corresponding overpotentials of 376 mV, 328 mV, 286 mV, 294 mV, 305 mV, and 312 mV (Figure 6b). The data indicate that the electrochemical efficiency of NiFe-LDH@selenized increases with higher levels of selenization. In particular, the NiFe-LDH@selenized-100 mg sample exhibited the lowest overpotential of 286 mV, which is 90 mV lower than that of the unmodified NiFe-LDH, reflecting a 24% reduction. Additionally, all selenized samples showed lower overpotentials than pure NiFe-LDH, indicating the beneficial effect of selenization on catalytic performance. Figure 6c presents the Tafel slopes for NiFe-LDH and NiFe-LDH/NiSe_2_/Fe_3_O_4_ composites, which were 94.1 mV/dec, 85.6 mV/dec, 63.6 mV/dec, 70.3 mV/dec, 74.6 mV/dec, and 76.9 mV/dec, respectively. The NiFe-LDH@selenized-100 mg sample had the smallest Tafel slope of 63.6 mV/dec, representing a 32.4% decrease compared to the unselenized NiFe-LDH (lowered by 30.5 mV/dec). The electrocatalytic kinetics of all selenized composites improved, evidenced by the lower Tafel slopes. Electrochemical impedance spectroscopy (EIS) studies revealed the charge transfer resistance of the materials, with the charge transfer resistances of the six samples being 76.0 Ω, 52.5 Ω, 37.3 Ω, 42.9 Ω, 56.9 Ω, and 59.7 Ω (see Figure 6d). Among them, NiFe-LDH@selenized-100 mg exhibited the lowest charge transfer resistance at 37.3 Ω, which is a 51% reduction compared to NiFe-LDH. The resistance of all selenized composites was lower than that of pure NiFe-LDH, further confirming that selenization can accelerate the reaction rate. Furthermore, the electrochemical active surface area (ECSA) was estimated by measuring the double-layer capacitance (Cdl) (see Figure S5), and the results indicated that selenization increased the number of active sites, thereby promoting more efficient electrochemical reactions. These findings are consistent with the observed decrease in overpotential and Tafel slope, confirming that NiFe-LDH@selenized outperforms NiFe-LDH in electrocatalytic performance. Overall, selenization not only improved the electronic transfer properties of the composite materials, but also enhanced their catalytic reaction kinetics. Additionally, we conducted a chronoamperometric stability test on the NiFe-LDH@selenized-100 mg sample (Figure 7). The results showed that the material maintained stable electrocatalytic performance after continuous operation for 60 h, with no significant decay, demonstrating excellent stability. In contrast, the unmodified NiFe-LDH could only operate stably for about 10 h [41], indicating that the stability of the material was significantly improved after selenium modification.

Table 1 shows a comparison of the catalyst we studied with other reported non-precious metal OER catalysts. The results indicate that the prepared NiFe-LDH@selenized-100 mg electrocatalyst outperforms existing high-performance non-precious metal catalysts in the OER, demonstrating its great potential for practical applications.

## 4. Conclusions

This study presents an efficient and straightforward method for selenizing NiFe-layered double hydroxide (NiFe-LDH) via hydrothermal synthesis, resulting in a cost-effective and high-performance catalyst for the oxygen evolution reaction (OER), NiFe-LDH@selenized. At a current density of 10 mA/cm^2^, the NiFe-LDH@selenized-100 mg exhibited an overpotential of only 286 mV, a Tafel slope of 63.6 mV dec⁻^1^, and a charge transfer resistance (Rct) of 37.3 Ω, representing reductions of 24%, 32.4%, and 51%, respectively, compared to pure NiFe-LDH. This remarkable OER electrocatalytic activity is attributed to enhanced electronic conductivity, increased concentration of high-valent metal species, and the formation of stable Ni-Se bonds, which provide more active sites and significantly enhance the catalytic efficiency of the composite. This simple synthesis method not only improves the electrochemical performance of the catalyst, but also offers advantages in terms of low cost and ease of implementation. Therefore, this strategy is expected to serve as a potential alternative material for electrocatalytic water splitting, and shows great promise for applications in clean energy conversion and catalysis.

## Figures and Tables

**Table 1:** Comparison table of different reported non-noble metal electrocatalysts with NiFe-LDH@Selenized-100 mg.

Electrocatalysts	Overpotential at 10 mA cm^−2^	Tafel Slope (mV dec^−1^)	Reference
CuO@CoV LDH	329 mV	65	[42]
RuO_2_-CeO_2_	350 mV	74	[43]
Ni-CAT/NiFe-LDH/CNFs	370 mV	79	[44]
s LDH	300 mV	90.2	[45]
Fe.Cu-LDH/ZIF-12	337 mV	89	[46]
NiFe-LDH@selenized-100 mg	286 mV	63.6	This work

## Data Availability

Data are contained within the article and Supplementary Materials.

## Supplementary Materials

The following supporting information can be downloaded at https://www.mdpi.com/article/10.3390/nano15040294/s1: Figure S1: Scanning electron micrographs (SEMs) of NiFe-LDH and NiFe-LDH@selenized: (a,b) NiFe-LDH@selenized-50 mg; (c,d) NiFe-LDH@selenized-150 mg; (e,f) NiFe-LDH@selenized-200 mg. Figure S2: (a) HAADF image and (b)–(f) MAPPING image of NiFe-LDH@selenized-100 mg. Figure S3: Energy-dispersive spectroscopy (EDS) point-scan image of NiFe-LDH@selenized-100 mg. Figure S4: X-ray photoelectron spectrum of NiFe-LDH@selenized-100 mg. Figure S5: Double-layer capacitance plot.
