# Supplementary material for: Enhancing Oxygen Evolution Catalysis by Tuning the Electronic Structure of NiFe-Layered Double Hydroxides Through Selenization
Source: Nanomaterials (Basel). 2025 Feb 14;15(4):294. doi: 10.3390/nano15040294 (PMC11857861; doi:10.3390/nano15040294)
Supplement: Supplementary file 1 [file nanomaterials-15-00294-s001.zip › nanomaterials-3467409-supplementary.pdf]

Enhancing Oxygen Evolution Catalysis by Tuning the Electronic Structure of NiFe-Layered Double Hydroxides Through Selenization

Ze Wang †, Yifang Liang †, Taifu Fang, Xinyu Song, Luobai Yang, Liying Wen, Jinnong Wang, Dongye Zhao * and Shifeng Wang *

Key Laboratory of Plateau Oxygen and Living Environment of Xizang Autonomous Region, College of Science, Xizang University, Lhasa 850000, China

* Correspondence: zdy@utibet.edu.cn (D.Z.); wsf@utibet.edu.cn (S.W.)

† These authors contributed equally to this work.

Supporting Information

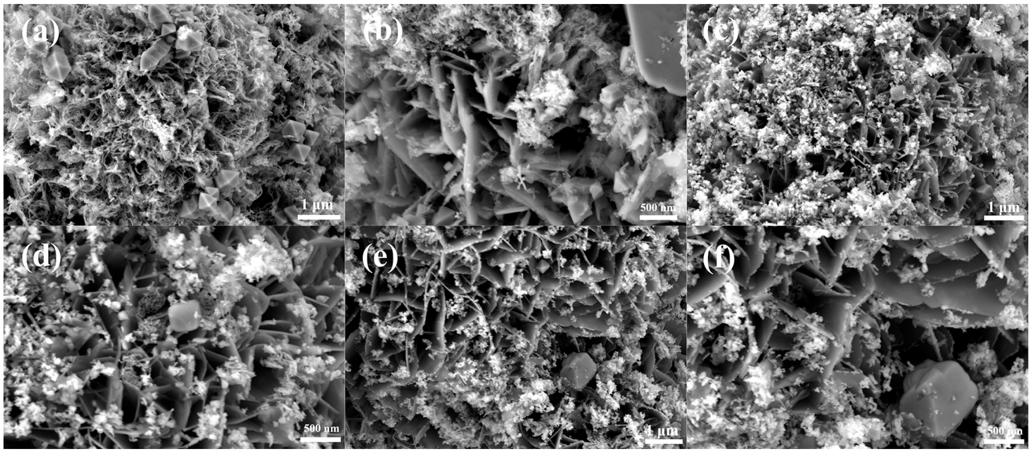

Figure S1. Scanning electron micrographs (SEMs) of NiFe-LDH and NiFe-LDH@selenized: (a,b) NiFe-LDH@selenized-50 mg; (c,d) NiFe-LDH@selenized-150 mg; (e,f) NiFe-LDH@selenized-200 mg.

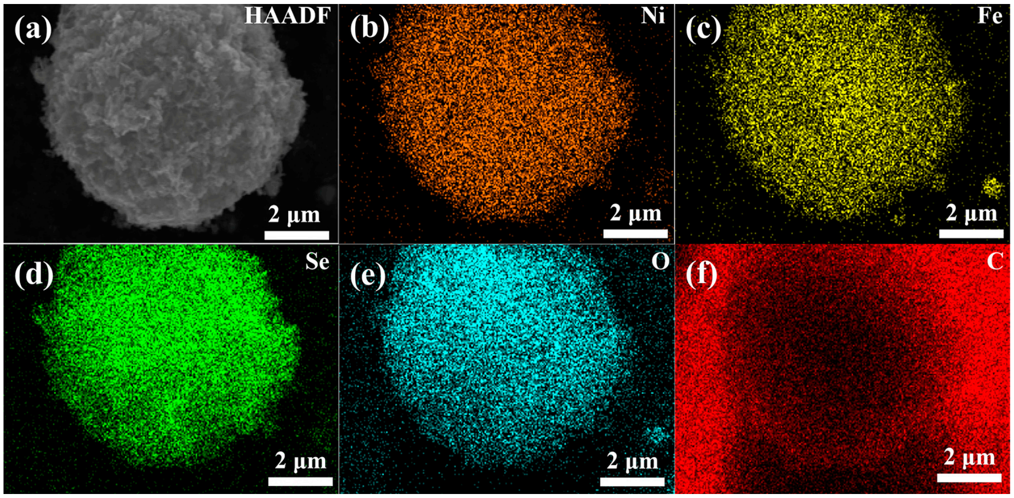

Figure S2. (a) HAADF image and (b)-(f) MAPPING image of NiFe-LDH@selenized-100 mg.

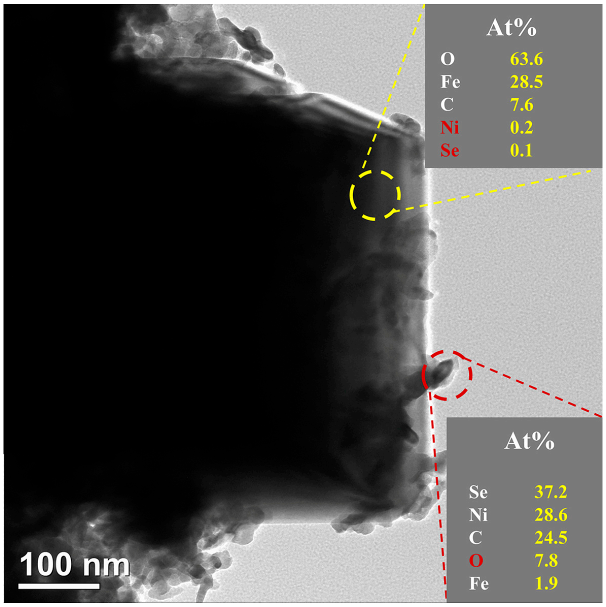

Figure S3. Energy-dispersive spectroscopy (EDS) point-scan image of NiFe-LDH@selenized-100 mg.

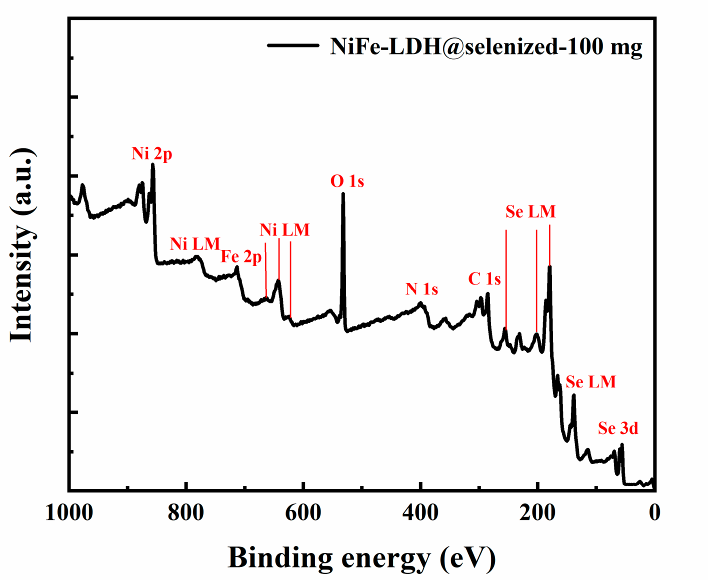

Figure S4. X-ray photoelectron spectrum of NiFe-LDH@selenized-100 mg.

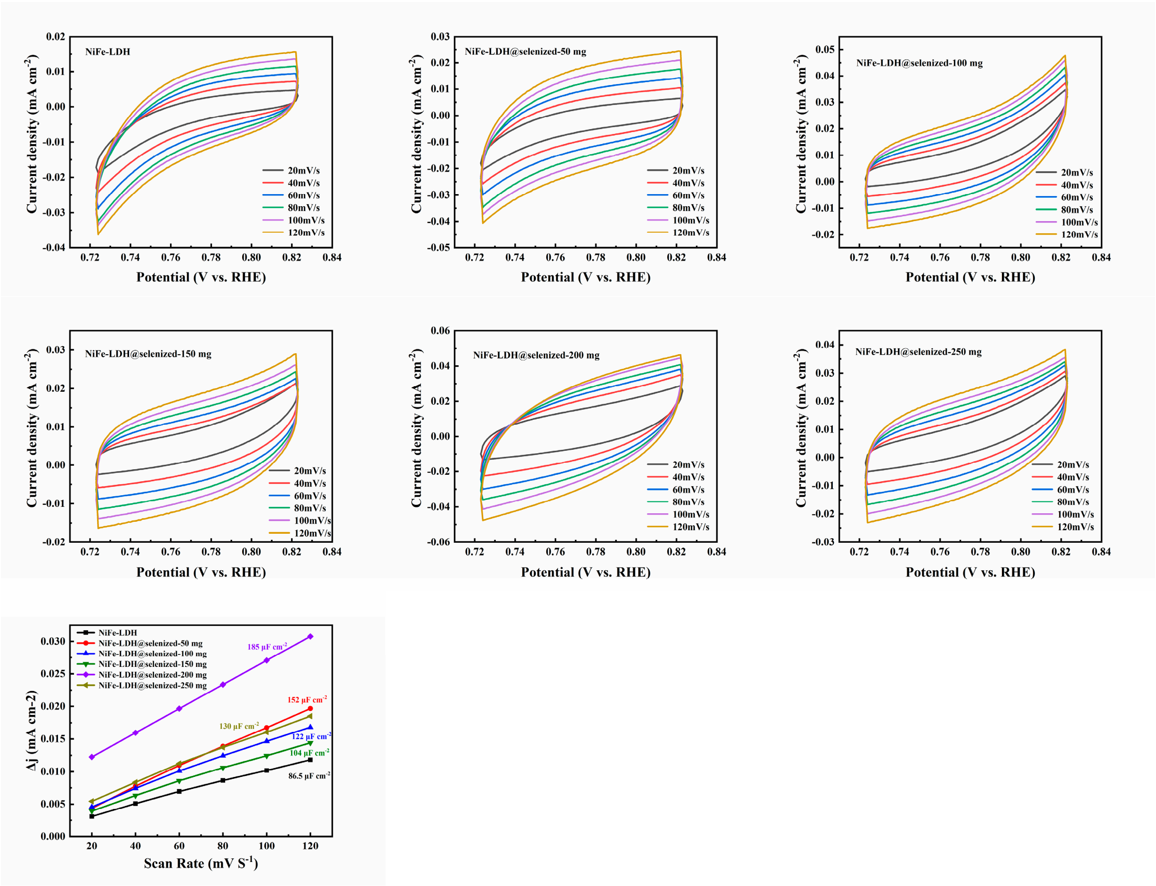

Figure S5. Double-layer capacitance plot.
